# Rheumatismal Pott’s Disease

**Published:** 1882-07

**Authors:** 


### RHEUMATISMAL POTT'S DISEASE.

Prof. Potain (*Jour. de Med. Pratique*) indicates a variety of Pott's disease, described in a thesis by M. Pouliot under the name of rheumatismal spinal disease, which admits of a more favorable prognosis than other varieties of the affection. The predisposition is produced by the arthritic diathesis, the spinal localization being induced by cold or violent efforts expended especially on vertebral articulations. Pains first occur in the spinal column, accompanied by irradiations, tingling, and convulsive movements of the limbs; then deformity of the spinal column is observed, consisting generally in anterior curvature, other rheumatic symptoms also occurring at the same time or alternately with the spinal symptoms. Paraplegia comes on gradually, but its progress is very slow, and abscess by congestion is exceptional. Resorted to in time treatment may be followed by excellent results, and consists chiefly in powerful revulsives, especially the actual cautery, accompanied by immobilization.—*Medical Times and Gazette.*

